# Hematoma of cavum septum pellucidum by ruptured aneurysm

**DOI:** 10.11604/pamj.2023.46.39.37692

**Published:** 2023-09-27

**Authors:** Fresnel Lutèce Ontsi Obame, Abad Chérif El Asri

**Affiliations:** 1 Department of Neurosurgery, Mohammed V Military Teaching Hospital, Rabat, Morocco

**Keywords:** Hematoma, septum pellucidum, aneurysm

## Image in medicine

A 57-year-old male patient, with no past medical history, was admitted with sudden-onset headaches, vomiting, and loss of consciousness. A neurological examination revealed disorientation. His pupils were equal and reactive to light. He had purposeful responses of all extremities. The blood pressure was 200/100 mmHg. Complete blood counts and coagulation profiles were normal. Unenhanced cerebral computed tomography showed corpus callosum hematoma, septum pellucidum hematoma, and intraventricular flooding corresponding to Fisher grade 4 subarachnoid hemorrhage. Cerebral angiography showed an anterior communicating artery aneurysm, suggesting the diagnosis of subarachnoid hematoma (SAH) due to a rupture of an anterior communicating artery aneurysm. An external ventricular shunt was placed. Afterward, the patient was treated by endovascular embolization the next day.

**Figure 1:**
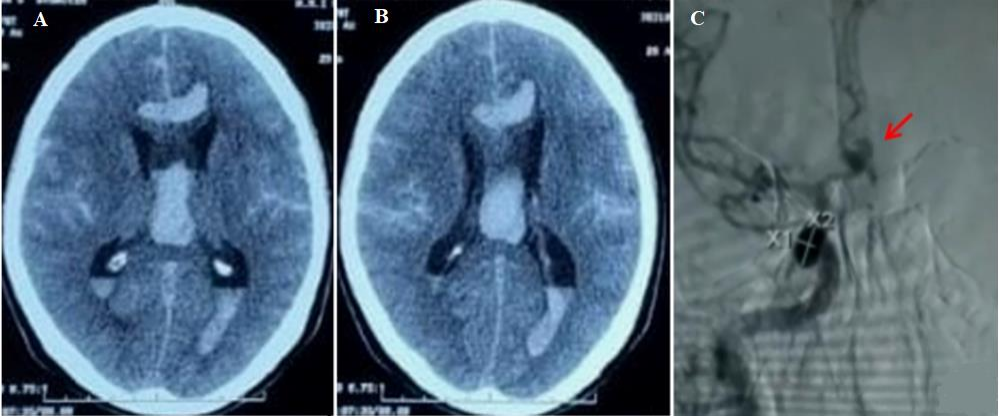
A,B) unenhanced cerebral computed tomography showing a hyperdensity in the subarachnoid space with corpus callosum hematoma, septum pellucidum hematoma and intraventricular hematoma; C) cerebral angiography showing an anterior communicating artery aneurysm, red arrow

